# Health status of people with intellectual disabilities: A systematic literature review: Ursula Griebler

**DOI:** 10.1093/eurpub/ckac130.032

**Published:** 2022-10-25

**Authors:** U Griebler, R Griebler, G Weber

**Affiliations:** 1 Department for Evidence-based Medicine and Evaluation, University for Continuing Education Krems, Vienna, Austria; 2 Cochrane Austria, University for Continuing Education Krems, Vienna, Austria; 3 Competence Centre for Health Promotion and Health Care, Austrian National Public Health Institute, Vienna, Austria; 4 Faculty of Psychology, University of Vienna, Vienna, Austria

## Abstract

**Background:**

People with intellectual disabilities (ID) have a right to the highest level of health and to non-discrimination in health care. There are an estimated 89,000 people with ID living in Austria. Little is known about their health status and about their health care. We carried out a systematic review with the aim to provide an overview of comparative studies on the health status and health behaviour of people with and without ID.

**Methods:**

The systematic literature search was conducted in 3 electronic databases for the search period 2008 to March 2020, supplemented by a reference list check and a search of relevant websites. Abstracts and full texts were dually screened, data extraction was performed by one person and checked by a second. Bias risk was assessed by two people using the AXIS tool for cross-sectional studies. Results were summarised narratively.

**Results:**

A total of 73 publications were included. The literature review reveals a very clear picture: people with ID have a shorter life expectancy, are more affected by illness and health problems, are more likely to have health-related limitations in daily living and are more likely to die from potentially preventable causes of death that could be avoided either through preventive measures or by high-quality medical care.

**Conclusions:**

To better assess the health situation of people with ID in Austria, Austria-specific data is needed. Furthermore, systematic initiatives in the areas of prevention, health promotion and health care are necessary. People with ID should therefore be systematically considered in all strategies and processes in the health sector. In terms of health literacy, a stronger information policy towards people with ID and towards their relatives and health care professions would be indicated.

**Key messages:**

- People with ID have a shorter life expectancy, more illness and health problems, health-related limitations in daily life and are more likely to die from preventable causes of death.
- Health data for people with ID is missing in Austria and systematic initiatives in the areas of prevention, health promotion and health care are strongly needed.

